# A machine learning analysis of correlates of mortality among patients hospitalized with COVID-19

**DOI:** 10.1038/s41598-023-31251-1

**Published:** 2023-03-11

**Authors:** Timothy B. Baker, Wei-Yin Loh, Thomas M. Piasecki, Daniel M. Bolt, Stevens S. Smith, Wendy S. Slutske, Karen L. Conner, Steven L. Bernstein, Michael C. Fiore

**Affiliations:** 1 Center for Tobacco Research and Intervention (UW-CTRI), University of Wisconsin School of Medicine and Public Health, 1930 Monroe St #200, Madison, WI 53711 USA; 2 Department of Medicine, School of Medicine and Public Health, University of Wisconsin, Madison, WI USA; 3 Department of Statistics, University of Wisconsin, Madison, WI USA; 4 Department of Educational Psychology, University of Wisconsin, Madison, WI USA; 5 Department of Family Medicine and Community Health, School of Medicine and Public Health, University of Wisconsin, Madison, WI USA; 6 Department of Emergency Medicine, Geisel School of Medicine at Dartmouth, Lebanon, NH USA

**Keywords:** Viral infection, Preventive medicine, Outcomes research, Risk factors

## Abstract

It is vital to determine how patient characteristics that precede COVID-19 illness relate to COVID-19 mortality. This is a retrospective cohort study of patients hospitalized with COVID-19 across 21 healthcare systems in the US. All patients (N = 145,944) had COVID-19 diagnoses and/or positive PCR tests and completed their hospital stays from February 1, 2020 through January 31, 2022. Machine learning analyses revealed that age, hypertension, insurance status, and healthcare system (hospital site) were especially predictive of mortality across the full sample. However, multiple variables were especially predictive in subgroups of patients. The nested effects of risk factors such as age, hypertension, vaccination, site, and race accounted for large differences in mortality likelihood with rates ranging from about 2–30%. Subgroups of patients are at heightened risk of COVID-19 mortality due to combinations of preadmission risk factors; a finding of potential relevance to outreach and preventive actions.

## Introduction

Numerous studies^1–3^ have identified premorbid risk factors for COVID-19 mortality: older age, male sex, and a history of certain comorbidities such as chronic renal disease or cardiovascular disease (see Supplementary Table 1 for recent studies on prediction of COVID mortality). Most of these studies have not examined vaccination status as a factor that might affect the nature or magnitudes of predictive factors. Also, many of the studies identifying such risk factors have used traditional multivariable analytic strategies^1–3^. However, alternative approaches such as machine learning (ML) methods have also been used to take advantage of their complementary strengths^4–12^. Such strengths include less strict assumptions about data distributions, more flexible approaches to missingness, ability to determine optimal and robust predictor cut-scores, heightened sensitivity to higher order interactions, and greater predictive accuracy across multiple prediction problems^13–15^. Of course, ML can have limitations as well such as overfitting or sensitivity to poor selection of training data.

ML may be particularly useful in predicting COVID outcomes using EHR data. This is because EHR data may produce challenges that are problematic for traditional multivariable analytic approaches such as linear or logistic regression. For instance, in the case of the current study using EHR data for prediction of mortality, the great number of predictors challenged the comprehensive evaluation of higher order interactions. Further, a meaningful number of variables had distributions that made it important to evaluate them within subgroups of the total sample. For instance, some insurance categories did not occur in certain age groups; while Medicare coverage occurred principally amongst older individuals, commercial insurance was essentially absent amongst such individuals. These examples involve regulatory and policy effects but naturally occurring variation also resulted in such nesting. For example, severe obesity was largely restricted to younger patients. In such cases of nested distributions, the effects of variables cannot be meaningfully estimated in certain groups of patients because of a lack of variation within the group. In addition, such distributions mean that analytic approaches that determine risk across an entire sample or population may substantially mis-estimate relations in some subgroups. While traditional analytic methods could be engineered ad hoc to address such issues, model specification and interpretation of model coefficients quickly becomes complicated as the number of predictor variables and their interactions increase. ML represents an efficient approach that can generate easily grasped and clinically informative predictive models in such circumstances.

As noted above, ML methods have been used previously to identify factors that predict COVID-19 severity. However, some of these studies have limitations that may have reduced the accuracy and generalizability of their results. For instance, many used fairly small and unrepresentative samples. A recent review of ML studies of COVID-19 mortality risk^16^ (see Supplementary Table 1) showed that many of the studies had samples that numbered only in the low thousands, or fewer, with samples often recruited from just a small number of health systems e.g., Refs.^5,6,8,17^. Also, few early studies were able to use vaccination status as a predictor so that its main and interactive relations with risk factors were unexplored. The current study employed an ML analytic strategy in a relatively large and diverse sample recruited from multiple sites with nationwide distribution; a sample for which COVID-19 vaccination history was known.

One goal of this study was to identify pre-hospital-admission patient characteristics that are relatively highly related to subsequent COVID-19 mortality: e.g., demographic variables and premorbid conditions that could have affected mortality amongst patients hospitalized with COVID-19 but that occurred prior to the development of severe disease. Such predictors index the risk of COVID-19 mortality in the absence of specialized testing or waiting until infection has occurred. In addition, we wish to demonstrate that predictors of COVID-19 mortality can be highly contextualized: i.e., varying meaningfully both with regard to other predictors and with regard to different healthcare sites included in the analyses. Thus, as other studies have done, we will show which predictors are generally most predictive of mortality across a sample and subsample. However, we will also demonstrate that the most relevant predictor variables that emerge can also be a function of site and other predictors.

Many prior ML studies that have focused on the prediction of COVID-19 mortality used predictors such as laboratory tests, COVID-19 symptoms and signs, post-hospitalization events such as ICU admission and use of mechanical ventilation e.g., Refs.^4–11^. Such variables are often highly predictive of COVID-19 outcomes such as death^12,16^ because they directly index severe COVID-19 (i.e., measures of disease severity predict severe disease). These likely inform post-infection clinical decision making but they require access to specialized information, which limits their public health reach. Moreover, their high predictive validity may mask or obscure the relations of other important variables that are more causally remote with regard to the ultimate COVID-19 outcomes.

It is important that additional research address inconsistencies in the literature regarding the factors that presage severe COVID-19. For instance, some studies have found Black race to be meaningfully related to COVID-19 mortality^2,4^, while other studies have not^3,18^. Similarly, the evidence is mixed as to whether some premorbid conditions such as hypertension are associated with COVID-19 severity^19–24^. This research can contribute to the evidence regarding such variables.

## Methods

This research uses supervised ML methods to explore the correlates of mortality in an entire sample of adult patients hospitalized for COVID-19 (N = 145,944) and a subsample of these patients (N = 86,732). The entire sample comprised admitted patients meeting COVID-19 criteria from February 1, 2020, through January 31, 2022; the subsample comprised a subset of those patients admitted from January 1, 2021 to January 31, 2022, a span during which COVID-19 vaccination was available.

### Study design

The COVID EHR Cohort at the University of Wisconsin (CEC-UW) is a retrospective cohort study funded by the National Cancer Institute (NCI). Healthcare systems from across the U.S. were invited to participate and 21 joined the cohort (Supplementary Fig. 1) and transferred data regularly to the CEC-UW Coordinating Center in Madison, Wisconsin. Each data transfer included new data on patients entering the cohort and any follow-up data from cohort members identified in prior data collection waves. All participating hospitals were nonprofit acute care facilities affiliated with academic medical centers.

### Ethics statement

The CEC-UW study was initially approved in May 2020 by the University of Wisconsin-Madison Health Sciences Minimal Risk Institutional Review Board (MR-IRB) for collection of de-identified EHR data. In February 2021, the MR-IRB approved a protocol change to a Limited Data Set. The MR-IRB also determined that the study met criteria for a human subjects research exemption and qualified for a waiver of informed consent under the Federal Common Rule. All participating health systems provided written notice of either their own institution’s IRB approval or determination of exemption status before sharing EHR data. In February 2021, the MR-IRB approved a change of protocol for a Limited Data Set, allowing the collection of additional information (e.g., death dates, five-digit zip codes) but excluding direct patient identifiers. Each patient in the data set from each health system was assigned an enduring cryptographically processed Patient ID based on the SHA256 algorithm, which yielded a 64-character unique and private hash-based message authentication code (HMAC). Study reporting follows STROBE guidelines^25^. All methods were carried out in accordance with relevant guidelines and regulations.

### Data collection

#### Extraction, harmonization, and secure transfer of EHR data

EHR data extraction code was created by programmers at UW School of Medicine and Public Health (Madison, WI), Yale New Haven Health (New Haven, CT), and Bluetree Network, Inc.^26^ (Supplementary Methods Text).

The extraction code was customized at each healthcare system to map to their EHR data to yield relatively uniform data sets. Additional data harmonization and quality assurance was done by CEC-UW staff (Supplementary Methods Text). Secure transfer of data from each of the 21 healthcare systems was accomplished via the transfer of data files to a secure SFTP (secure shell [SSH] File Transfer Protocol) portal located at the UW-Madison CEC-UW Coordinating Center.

#### Extracted data categories

Each healthcare system transferred five source data files with patient- and encounter-level information on: (1) sociodemographic and health characteristics; (2) pre- and post-COVID-19 ICD-10 diagnoses; (3) clinical encounter data including treatment site (e.g., inpatient, outpatient), encounter-based ICD-10 diagnoses, mortality, ICU admission, intubation, and other clinical data; (4) selected laboratory test results linked to encounters; and (5) selected medications linked to encounters. Not all these data were used in the present analyses given their intended focus. Healthcare systems provided data only for closed clinical encounters; inpatient encounters were closed via discharge or death. Data on post-discharge outcomes or treatment or outcomes at nonparticipating healthcare systems were not captured.

### Analysis sample

The analysis samples comprised 145,944 (full sample) and 86,732 (subsample) adult patients hospitalized with COVID-19 who were admitted to a participating hospital and completed their hospitalization over the periods from February 1, 2020 to January 31, 2022 (full sample) and from January 1, 2021 to January 31, 2022 (subsample). Analysis sample inclusion criteria included: (1) ≥ 18 years old; (2) the inpatient encounter was the first COVID-19 hospitalization with duration ≥ 24 h (or, if < 24 h, admission to ICU or death during the hospitalization); (3) COVID-19 ICD-10 diagnosis (U07.1 or J12.82) during the hospitalization or positive COVID-19 PCR test result in a 14-day window (± 7 days centered at the admission date); and (4) prior contact with the health system to permit extraction of pre-COVID-19 ICD-10 diagnoses to calculate the Elixhauser Comorbidity Score^27^. For the full sample, 74.0% (n = 107,960) had both a positive PCR test result and a COVID-19 ICD-10 diagnosis, 5.7% (n = 8367) had only a positive PCR test, and 20.3% (n = 29,617) had only a COVID-19 ICD-10 diagnosis at the time of hospitalization. For the subsample, 75.2% (n = 65,192) had both a positive PCR test result and a COVID-19 ICD-10 diagnosis, 5.4% (n = 4706) had only a positive PCR test, and 19.4% (n = 16,834) had only a COVID-19 ICD-10 diagnosis at the time of hospitalization.

### Primary outcome

The primary and sole outcome for these analyses was in-hospital mortality during the index COVID-19 hospitalization documented via EHR.

### Non-outcome variables

Patient-level variables include age (at time of entry into the cohort), sex, race, ethnicity, body mass index (BMI), insurance status, Elixhauser Comorbidity overall score and constituent item scores (Supplementary Table 2), Rural/Urban Commuting Area (RUCA) code groups, Social Deprivation Index score (SDScore), and vaccination status. Preadmission vaccination status was coded as binary (no vaccination versus any vaccination) and by the number of vaccine doses (0, 1, 2 or 3 doses). Supplementary Table 3 presents the types of vaccines that patients received for their first, second, and third vaccinations. Patients are considered ‘unvaccinated’ in the absence of an EHR record of vaccination. Patients aged ≥ 90 years were coded as 90 at the time of data extraction. See Table 1 for data on age, race, ethnicity, BMI categories, insurance status, vaccination status, RUCA, SDScore, and Elixhauser score for the full sample. Such data are presented in Supplementary Table 4 for the subsample. Race and ethnicity categories were based on definitions used by the National Institutes of Health^28^. The Elixhauser Comorbidity Score was calculated using van Walraven weights^27^ based on ICD-10 diagnoses (present vs. absent) determined via a 5-year look back pre-COVID-19. RUCA and SDScore were derived based upon the patient’s ZIP code and were determined for the patient’s aggregated ZIP code tabulation area (ZCTA). Supplementary Table 5 lists sites by number and the sample size associated with each site (healthcare system: not identified by name).

**Table 1:** The characteristics of the full sample averaged across patients from all health systems (N = 145,944) and including status on covariates and vaccination variables.

Patient characteristic	*N*	%	*M*	SD
Elixhauser comorbidity index			5.71	9.78
Age (years)			61.13	18.39
Social deprivation score			53.14	31.10
Age groups				
Under 60 years	61,685	42.3		
60–70 years	33,540	23.0		
Over 70 years	50,719	34.8		
Sex				
Female	74,538	51.1		
Male	71,402	48.9		
Other	4	0.00		
Race				
American Indian/Alaska Native	546	0.4		
Asian	3882	2.7		
Black or African American	34,663	23.8		
Native Hawaiian or other pacific islander	588	0.4		
White	85,851	58.8		
Other race	17,384	11.9		
More than one race	571	0.4		
Missing	2459	1.7		
Ethnicity				
Not Hispanic or latino	120,761	82.7		
Hispanic or latino	22,373	15.3		
Missing	2810	1.9		
Body mass index				
Underweight	4504	3.1		
Healthy weight	33,608	23.0		
Overweight	41,473	28.4		
Obese	48,138	33.0		
Severely obese	16,627	11.4		
Missing	1594	1.1		
Insurance status				
Medicare	75,961	52.0		
Medicaid	17,419	11.9		
Commercial	38,728	26.5		
Uninsured	3836	2.6		
Other	10,000	6.9		
Rural–urban commuting area				
Rural	2410	1.7		
Small town	4640	3.2		
Micropolitan area	9570	6.6		
Metropolitan area	129,221	88.5		
Missing	103	0.1		
Vaccination status				
No recorded vaccination	123,126	84.4		
Yes, at least one	22,818	15.6		
Vaccination doses				
0	123,126	84.4		
1	5589	3.8		
2	13,651	9.4		
3	3578	2.5		

## Statistical analysis

### Descriptive statistics and missingness

Descriptive statistics for the analysis sample characteristics and selected outcome analyses were computed using SPSS version 27 (IBM Corp) and R version 4.1.2 (R Foundation for Statistical Computing). There were no missing data for the primary outcome. Missing data for covariates are reported in Table 1 and Supplementary Table 4. The data sets have missing values in 4 categorical variables (Race, Ethnicity, BMI, RUCA) and 1 continuous variable (SDScore). Missing values in each categorical variable were recoded as “Unknown” and entered as unknown or missing variables in the machine learning analyses. This leaves SDScore as the only variable with missing values.

### Machine learning analyses

The primary ML approach used to generate decision trees and importance scores was GUIDE^29–31^. GUIDE is a ML algorithm for building classification and regression tree models by recursively partitioning the data. In this report, the response variable Y is binary-valued (Y = 1 if died, Y = 0 if alive) and least-squares regression trees are used. At each node of a tree, the observations are divided into two subsets by a split of the form “X ≤ c” (if X is an ordinal variable) or “X ∈ A” (if X is a categorical variable), where X is the variable with the most significant contingency table chi-squared test of X (columns) versus the values of Y (rows). If X is an ordinal variable (such as Age), its values are grouped into 3 or 4 intervals at the sample quantiles to form the columns of the table. If X is a categorical variable, its categories are used to form the columns. If X has missing values, an additional column for missing values is added to the contingency table. After the most significant X is found, a search is carried out for the split of the data based on the observed values of X that minimizes the sum of squared deviations of the Y values around each node mean. If X is ordinal, the search is over the sets {X = NA}, {X ≤ c and X = NA}, or {X ≤ c and X ≠ NA}, where c ranges over the midpoints of consecutively ordered values of X, and NA denotes the missing value code. If X is categorical, the search is over all subsets A of the categories (including the NA category, if applicable) of X. The split procedure is repeated recursively on each node until an overly large tree is obtained. Then it is pruned to a smaller size to maximize a tenfold cross-validation estimate of prediction accuracy. Importance scores reflect the total chi-square associations of variables with mortality up to 4th-level interactions. Missing values in predictor variables are not imputed. At each split of a node, GUIDE determines whether missing values are sent to the left or the right branch based on model fit.

GUIDE has compared well with other methods in terms of producing solutions that generalize to new data: e.g., when compared with Lasso, stepwise regression, multivariate adaptive regression splines, support vector machine, random forest, and Rpart and M5 generated solutions (REFS^32–34^). A manual for GUIDE can be found at: http://www.stat.wisc.edu/%7Eloh/treeprogs/guide/guideman.pdf, which also provides access to downloadable software. For more information on GUIDE see the Supplementary Methods Text.

Different decision trees were developed using different samples and variables to explore the robustness or stability of the findings. One set of trees was developed from data over the whole study period (February 1, 2020–January 31, 2022: full sample analyses) while another set was based on only the second year of the study period (January 1, 2021 to January 31, 2022: subsample analyses). These two sets of analyses contrasted solutions obtained for time spans that likely differed in multiple ways: the COVID-19 variants that were prevalent in the different periods^35^, the adoption of different patient management and treatment methods, and the availability of vaccines (primarily occurring only after January 2021). Thus, the subsample analyses serve as sensitivity tests with regard to the full sample analyses. In addition, both the full sample analyses and the subsample analyses were done with and without site (healthcare system) being entered as a predictor. A key feature of this work is its capacity to accommodate predictive effects that are nested, meaning the effect is best understood in the context of other predictive variables. Site is entered into these models as one indicator of this. Solutions are also obtained without site effects since these may best reflect predictor-outcome relationships in applications where healthcare systems cannot be matched with the particular healthcare systems participating in this research (i.e., predictions are based on the substantive predictors per se rather than on predictions nested within sites). Also, leaving site out of the models is another way of showing the importance of site related differences in terms of predictor-mortality associations.

## Results

### Characteristics of the sample and mortality and vaccination rates

The characteristics of the full sample averaged across patients from all healthcare systems are depicted in Table 1, which lists status on covariates and vaccination variables. Nearly all vaccinations (99.9%) occurred in the second year of the study period: i.e., from January 2021 to January 2022.

### Decision trees

#### Full sample

**Figure 1:**
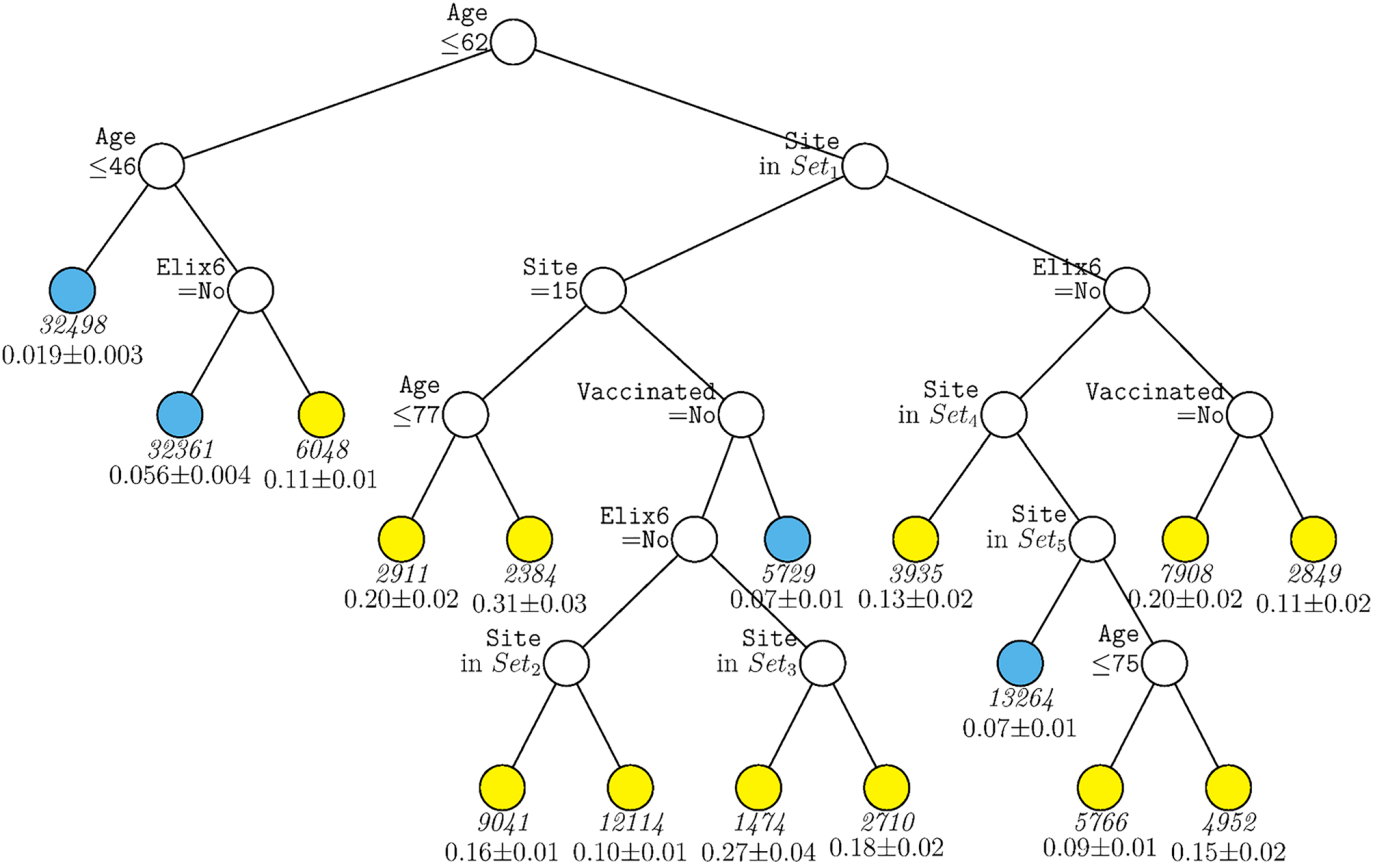
GUIDE subgroup model for differential outcomes for the Full sample. At each split, an observation goes to the left branch if and only if the condition is satisfied. *Set*_1_ = {Site 2, Site 4, Site 5, Site 7, Site 8, Site 13, Site 14, Site 15, Site 18}. *Set*_2_ = {Site 2, Site 5}. *Set*_3_ = {Site 5, Site 13}. *Set*_4_ = {Site 9, Site 16, Site 19}. *Set*_5_ = {Site 1, Site 6, Site 12, Site 17}. Sample sizes (in italics) and 95% simultaneous confidence intervals for mortality rate printed below nodes. Terminal nodes with means above and below overall mortality rate of 0.089 are colored yellow and skyblue respectively.

Figure 1 displays the decision tree generated for the full sample including site as a predictor. The tree was pruned from a larger tree with 70 terminal nodes to optimize a tenfold cross-validation estimate of prediction error. This figure shows that age was the variable selected as having the strongest relations with mortality over all other predictors, with those over age 62 years having generally higher mortality rates (indicated by yellow terminal nodes). Amongst those under age 62, only a further split on age and past-year uncomplicated hypertension (Elixhauser item 6) contributed significantly to prediction after pruning. For persons under age 46, mortality rates were quite low (2%). Results showed that many more variables survived pruning and significantly predicted mortality amongst those over 62 years of age. Site appeared in many arms of the tree for such individuals (see Supplementary Table 5 for site n’s). As such healthcare system or hospital matters especially for older patients although healthcare system may also code for factors correlated with it. Other variables contributing to prediction in this branch were uncomplicated hypertension, vaccination, and further splits on age. Thus, this tree shows relatively strong associations of age, hypertension, vaccination, and site with mortality but with site and vaccination showing significant relations only amongst older patients. Depending on the terminal nodes, mortality rates varied from about 2 to 31%.

**Figure 2:**
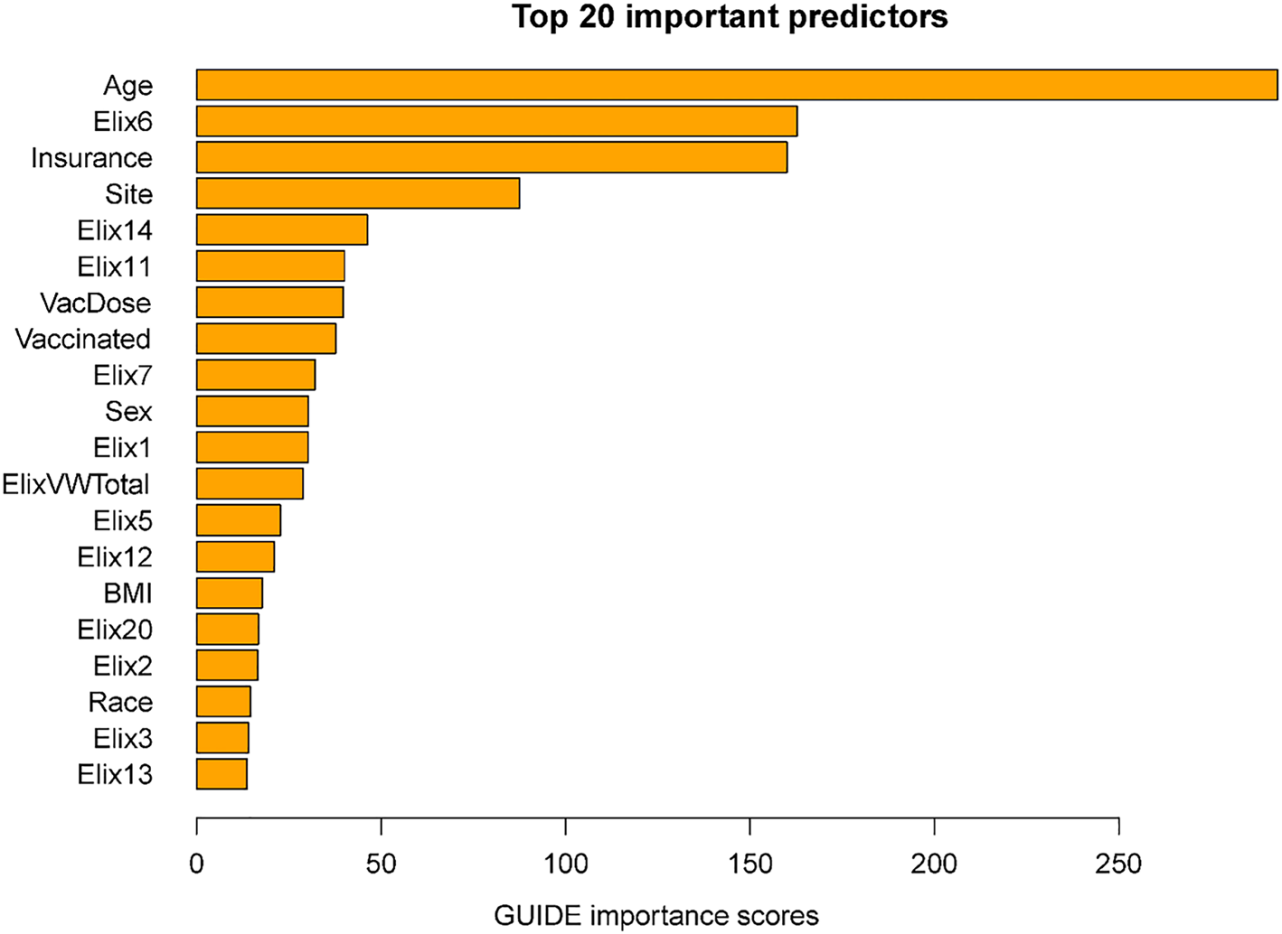
Twenty most important variables and their GUIDE importance scores for predicting mortality from full sample. *Elix6* hypertension, uncomplicated, *Elix14* renal failure, *Elix11* diabetes, uncomplicated, *VacDose* number of vaccine doses, *Vaccinated* any vaccine dose (vs. none), *Elix7* Hypertension, complicated. *Elix1* Congestive heart failure, *ElixVWTotal* weighted comorbidity total score, *Elix5* peripheral vascular disorders, *Elix12* diabetes, complicated, *Elix20* solid tumor without metastasis, *Elix2* cardiac arrhythmias, *Elix3* valvular diseases, *Elix13* hypothyroidism.

Importance scores reflect the magnitude of association of predictors via both their main effects and interactions. Some variables might have had significant associations with mortality but were not included in the decision trees since their chi-square values were only slightly less predictive than the included variables. The importance scores reflect the overall contributions of such variables. Figure 2 shows the 20 highest importance scores of the predictors in the full sample model that includes site. This figure shows that the variables that entered the decision trees achieved high importance scores: e.g., age, hypertension, vaccination, and site. Other variables with high importance scores were insurance coverage, sex, and a variety of comorbidities such as renal failure, diabetes, and others (see Fig. 2 caption). Supplementary Table 6 shows the mortality rates associated with the different insurance categories (these do not reflect interactions of insurance with other variables).

**Figure 3:**
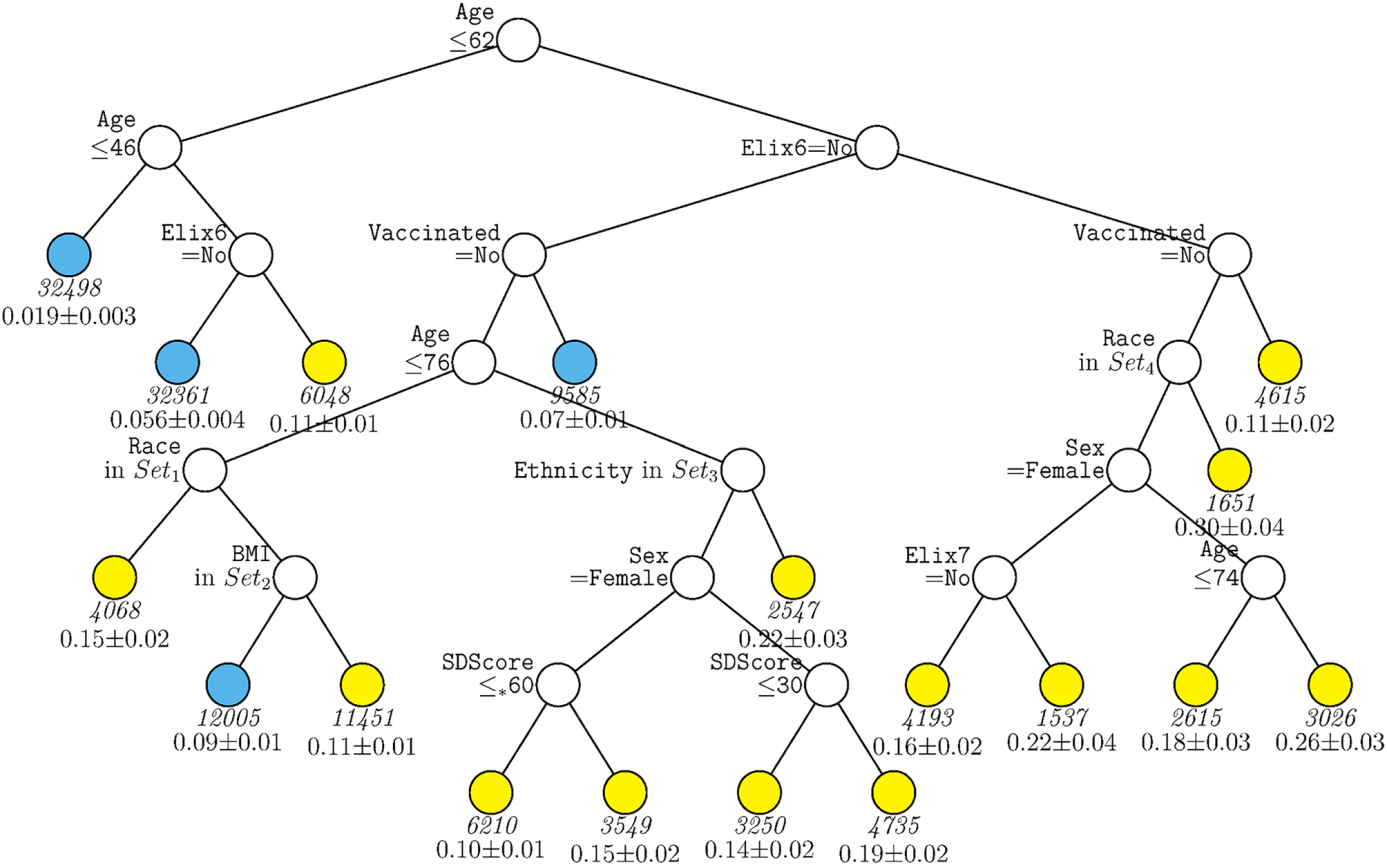
GUIDE subgroup model for differential outcomes for the Full sample, without Site. At each split, an observation goes to the left branch if and only if the condition is satisfied. Symbol ‘</=_*_’ stands for ‘C or missing’. *Set*_1_ = {American Indian or Alaska Native, Asian, Other Race Not Specified, Unknown, Not Reported, or Missing}. *Set*_2_ = {Healthy Weight. Overweight}. *Set*_3_ = {Not Hispanic or Latino}. *Set*_4_ = {Black or African American, Native Hawaiian or Other Pacific Islander, White}. *Elix6* hypertension, uncomplicated, *Elix7* hypertension, complicated, *SDScore* social deprivation score. Sample size (in italics) and mean of Mortality printed below nodes. Terminal nodes with means above and below value of 0.089 at root node are colored yellow and skyblue respectively.

A second decision tree analysis was conducted with the same predictors in the full sample excepting site. This tree (Fig. 3) shared features with the ‘site tree’; age, hypertension (both complicated and uncomplicated in this tree), and vaccination remained significant predictors. However, the absence of site allowed other predictors to account for significant differences in mortality likelihood. These variables included sex, race, ethnicity, BMI, and social deprivation score. Higher mortality rates were associated with male sex, lack of vaccination, higher social deprivation, and Hispanic ethnicity. A key observation is the nested nature of the associations. For example, different racial groups (e.g., American Indian/Alaskan Native, Asian) predicted mortality risk especially well in those over age 62 but not those under age 62. The association of vaccination was especially strong in those over 62 years of age. Sex was especially predictive of mortality amongst those who were unvaccinated versus vaccinated. Additionally, higher social deprivation scores (i.e., greater deprivation) were especially predictive of mortality amongst those over age 76.

#### Subsample

**Figure 4:**
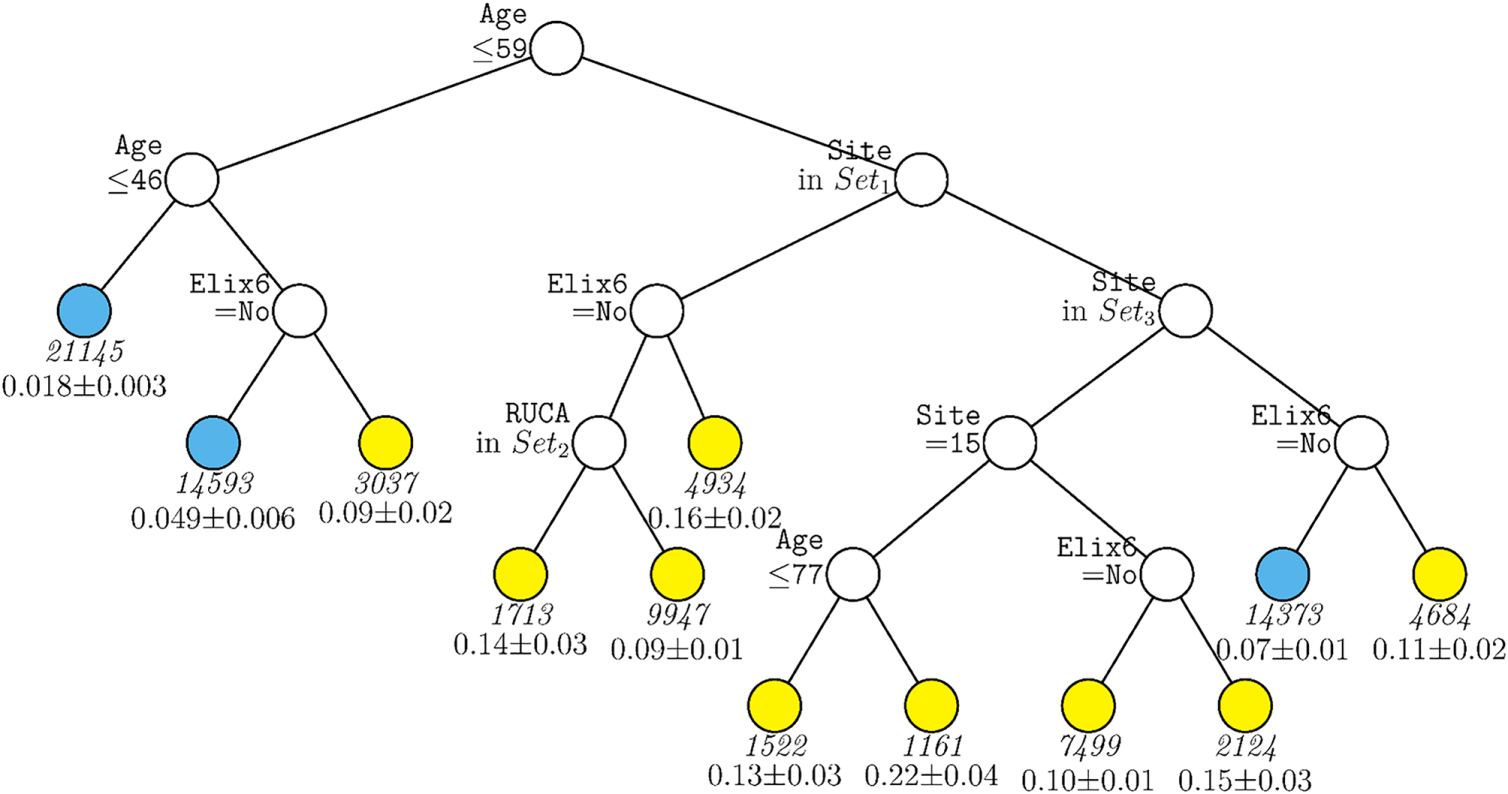
GUIDE subgroup model for differential outcomes for the Subsample. At each split, an observation goes to the left branch if and only if the condition is satisfied. *Set*_1_ = {Site 1, Site 3, Site 7, Site 9, Site 10, Site 12, Site 19, Site 21}. *Set*_2_ = {Micropolitan Area, Rural}. *Set*_3_ = {Site 2, Site 5, Site 11, Site 15, Site 16}. Sample sizes (in italics) and 95% simultaneous confidence intervals for mortality rate printed below nodes. Terminal nodes with means above and below value of 0.073 at root node are colored yellow and skyblue respectively.

The same two decision tree models were run with the subsample that comprised only patients who had been hospitalized during the period from January 1, 2021 to January 31, 2022 when vaccines were available. The model including site (Fig. 4) comprised some of the same variables as did the full sample model with site: age, site, and hypertension. However, the cut-scores for age were somewhat different, this tree included RUCA but not vaccination status, and the sites that differentiated node splits differed as well.

The subsample analysis without site identified very similar predictors as were identified in other analyses (Supplementary Fig. 2). As such, mortality rates tended to be higher with advanced age, a history of hypertension, and in the unvaccinated.

**Figure 5:**
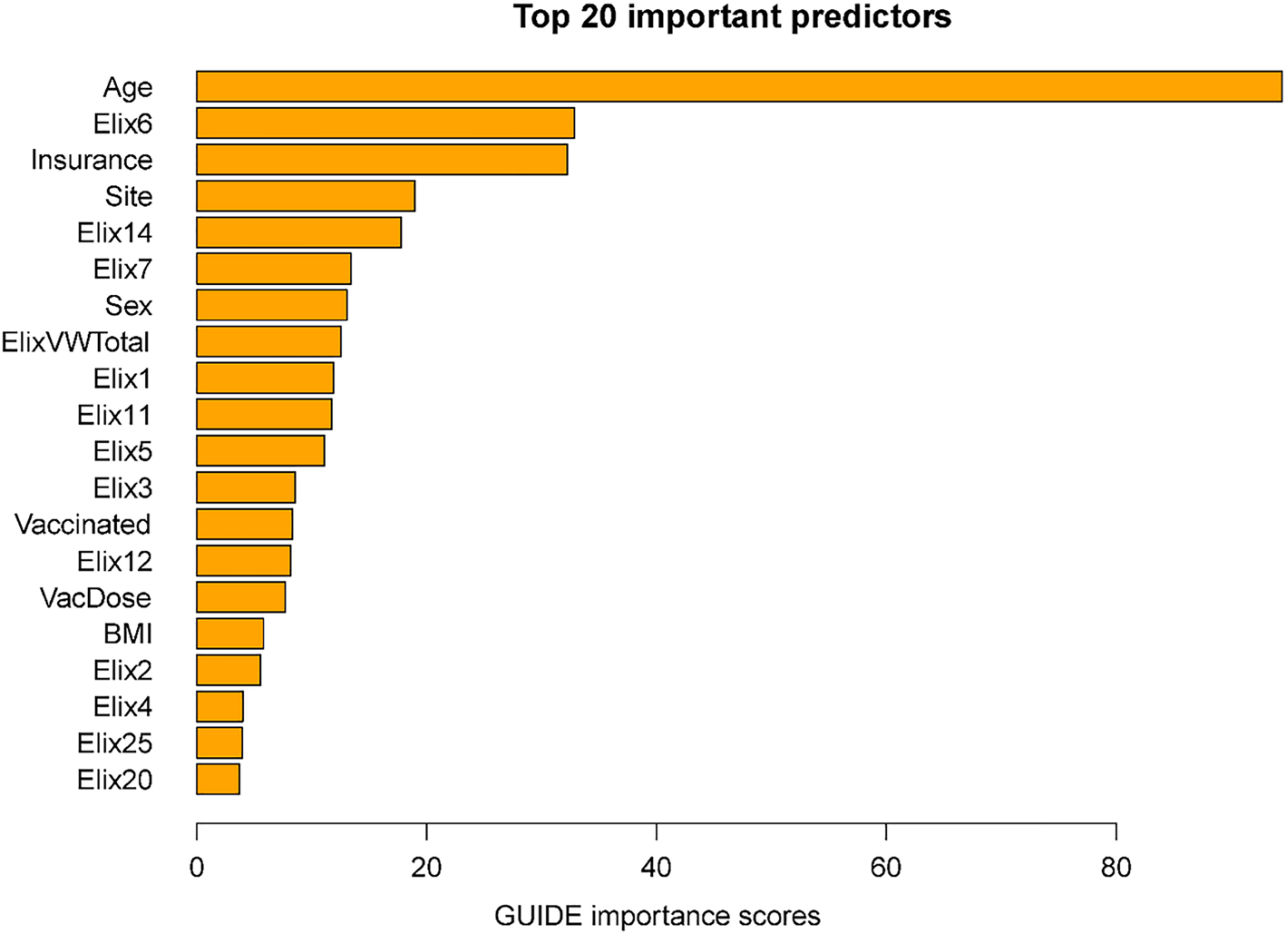
Twenty most important variables and their GUIDE importance scores for predicting Mortality from Subsample. *Elix6* hypertension, uncomplicated, *Elix14* renal failure, *Elix7* Hypertension, complicated, *ElixVWTotal* weighted comorbidity total score, *Elix1* congestive heart failure, *Elix11* diabetes, uncomplicated, *Elix5* peripheral vascular disorders, *Elix3* valvular diseases, *Vaccinated* any vaccine dose (vs. none), *Elix12* diabetes, complicated, *VacDose* number of vaccine doses, *Elix2* cardiac arrhythmias, *Elix4* pulmonary circuation disorders, *Elix25* fluid and electrolyte disorders, *Elix20* solid tumor without metastasis.

Figure 5 displays the importance scores for the subsample including site. In general, this list shows good correspondence in terms of the predictors identified in the full sample. Perhaps the biggest difference is the smaller magnitude of the relation of vaccination with mortality in the subsample than in the full sample. This may reflect the fact that vaccination and time (i.e., vaccine availability) are confounded in the full sample analyses. Thus, vaccination in these models may partly act as a proxy for hospitalizations occurring long after the initial phase of the pandemic when case mortality rates were especially high.

## Discussion

This research used ML strategies to explore the associations of demographic and comorbidity risk factors with mortality in a large sample of patients hospitalized with COVID-19 in healthcare systems distributed across the United States. The analyses identified risk factors that have especially strong relationships with mortality and demonstrate how such risk factors interact in predicting mortality. The 10 risk factors with the strongest overall associations with mortality, reflecting both their main and interactive effects, were age, uncomplicated hypertension, insurance status, site (health system), renal failure, diabetes, vaccination status (binary and number of immunizations), complicated hypertension, and sex.

Most of the risk factors listed above have been implicated in COVID-19 severity in past research (e.g., Refs.^1–3^). However, the present study makes several contributions. First, it was conducted in a particularly large sample comprising patients from multiple healthcare systems across the United States. Second, it exclusively explored variables that captured COVID-19 risk factors that preceded COVID-19 infection and that do not index infection severity once contracted. Such variables are highly relevant to the level of pre-infection risk of COVID-19 mortality if contracted; such data can be used in estimating mortality risk prior to intensive laboratory assessments or waiting for the disease to require progressively more intense intervention (such as intubation). This information can also be used in public health outreach and education efforts and by emergency department physicians to ascertain risk. Third, this research provides strong evidence that risk is meaningfully nested within patient subgroups, suggesting that data on population-wide risk relations may not optimally capture risk for many patients.

This research was unusual in showing particularly strong associations between hypertension and mortality. Prior research had produced a mixed pattern of association between these variables^19–23^ with some research reporting a significant relationship^36–38^ while other research did not^39,40^. This mixed pattern of evidence has led to conflicting pronouncements regarding hypertension risk by authoritative groups^41^. Some research suggests that the mixed evidence concerning hypertension risk can be attributed to its association with comorbidities such as other cardiovascular disease or with age^41,42^. This is consistent with evidence that the association of hypertension with COVID-19 severity is sometimes reduced by statistical control of covariates^43^. Thus, in a study of 17 million National Health Service patients, Williamson et al.^39^ found that the heightened risk of COVID-19 mortality related to hypertension was largely accounted for by hypertension’s association with diabetes and obesity. In contrast, our results show that uncomplicated hypertension and not complicated hypertension, was especially strongly related to COVID-19 mortality. Moreover, both the decision trees and the importance scores suggest that it was particularly highly associated with mortality relative to other comorbidities or obesity and was not restricted to a particular age group. Finally, while we did not control for the use of hypertensive medication in the current analyses, prior research suggests that such medication per se does not significantly affect COVID-19 severity^37,41,44,45^.

While Black race has been found to be associated with more severe COVID-19 outcomes in some studies and populations^2,4^, there was little evidence of this in this research. In fact, the decision tree without site (Fig. 3) showed that Black race was associated with an arm that conferred lower risk (along with other races).

The importance scores show good consistency regarding the findings in analyses of the full sample versus the subsample, which included only patients hospitalized in the second year of the study. This consistency was obtained despite factors that likely changed over the two time periods, factors such as new COVID-19 variants (to the extent that their prevalence varied with the two contrasted time frames^35,46,47^ the advent of effective vaccines, and advances in treatment or management practices over the course of the study^48^).

There were numerous examples where predictors exhibited detectable effects in certain subgroups but not others. For instance, vaccination, sex, BMI, and race were significantly associated with mortality only in older patients (see Figs. 1, 3). Further, the strength of associations of numerous predictors were dependent on site (e.g., vaccination, comorbidity, age: Fig. 1). Thus, while prior research showed that vaccination reduces the risk of mortality in hospitalized patients^49^, the current research shows that such risk reduction depends not only upon site but also on the age of the patient and comorbidity status. The current research cannot reveal why site was so highly associated with mortality. Sites differed in many ways including treatments and management strategies used, additional uncontrolled patient characteristics, and timing of disease surges. The current research does not permit strong inference regarding these factors.

The cumulative contributions of the various risk factors via their interactive and non-interactive associations identified groups that diverged dramatically in their mortality rates. For instance, Fig. 1, depending upon their status on the variables of age, hypertension, race, and vaccination status, some groups had a mortality rate of 2% while other patients had a mortality rate of 30%.

This research did identify risk factors that were highly associated with COVID-19 mortality across the entire sample: e.g., age, insurance status, and hypertension. Hypertension not only had a high importance score (Fig. 2) but was the rare variable that was significantly predictive across most age groups (Figs. 1, 4). With regard to insurance status, patients on Medicare and the uninsured were clearly at elevated risk for death (Supplementary Table 6). Insurance status may not have entered any decision tree because age was selected over Medicare status during pruning. However, it is important to note that most of the variables that had high importance scores as computed over the full sample, also had effects that varied significantly as a function of other risk factors. Thus, these analyses suggest that greater understanding of the risk for COVID-19 related mortality would be achieved if such relations were examined in subpopulations since the relations of numerous risk factors with mortality vary meaningfully as a function of other risk factors.

In sum, these results revealed variables that were important predictors across both the full sample and the sub-sample and when site effects were and were not taken into account. These variables had high importance scores in the two samples (e.g., age, hypertension, sex, renal failure, congestive heart failure, vaccination). However, this research also shows that predictive relations can differ meaningfully when used with different sites and populations as indicated by the numerous and large magnitude site effects as seen in the regression trees. Thus, in this research, instead of attempting to derive prediction models in wholly separate patient or site populations (e.g., with training and test samples), we opted to show how different sites and populations affected predictor-outcome relationships. Such variability in predictive relationships needs to be considered when attempting to generalize results to any particular healthcare setting or population.

The findings of this research might be used in policies aimed at outreach and prevention efforts. For instance, the age-related association of vaccination with decreased mortality might be used in outreach that encourages greater vaccination in older patients. The effect of vaccination in patients over 62 years of age and who had hypertension is particularly striking. Depending on status on other factors such as site, vaccination was associated with mortality rates that were often half of those of unvaccinated patients (Fig. 1). Outreach efforts might especially encourage vaccination amongst those who have hypertension given its strong association with mortality in such patients. Finally, the powerful findings associated with site^50^ encourage further exploration of the factors that can account for such effects. Such site effects, however, also show the constraints in generalizing findings to other patients and healthcare settings.

Limitations of this work include the fact that mortality rates reflect all-cause mortality; some deaths may have occurred for reasons other than COVID-19 infection. Deaths outside of the healthcare systems and that occurred post-discharge were not available. Also, the analysis sample did not comprise any non-hospitalized patients. No doubt, different associations would have been obtained if persons with a broader range of COVID-19 severity had been included. The associations of risk factors with mortality would also certainly change if post-admission events such as symptoms or test results were included as predictors^12^. Additionally, data on hospital features and care and staffing patterns at hospitals were unavailable and therefore site effects could not be further explored. Also, data were not available on the type of COVID-19 variants infecting patients and we did not compare different vaccines in terms of their relations with mortality. Further, we did not use a design in which we derived a prediction model and then validated it in a new sample of subjects. We did not use this strategy since we believed that use of the whole sample with tenfold cross-validation would yield the most accurate data on associations with COVID-19 mortality and because we wished to demonstrate the influence of different sites and patients on the nature of observed relationships. Moreover, the ML strategy we used might not have been an optimal approach relative to other strategies such as ensemble methods^12^. Finally, the study sample comprised only COVID-19 infected patients and no non-infected control patients.

## Supplementary Information

Supplementary Information.

## Data Availability

The existing Data Transfer and Use Agreements negotiated with each of the participating healthcare systems preclude the University of Wisconsin from sharing CEC-UW data with any entity at this time. Information Management Services, Inc. (IMS), under contract with the National Cancer Institute (NCI), is responsible for housing the final CEC-UW dataset. A small number of healthcare systems have put limits on the extent of data sharing. Data from most healthcare systems will eventually be made available to approved researchers, who are to be determined by NCI and/or IMS. The datasets generated and/or analyzed during the current study are not publicly available because they have not yet been transferred to the NCI contractor Information Management Services, Inc., (where they will be available after February 1, 2023) but are available from the corresponding author on reasonable request.
