# Supplementary material for: A machine learning analysis of correlates of mortality among patients hospitalized with COVID-19
Source: Sci Rep. 2023 Mar 11;13:4080. doi: 10.1038/s41598-023-31251-1 (PMC10007654; doi:10.1038/s41598-023-31251-1)

**SUPPLEMENTARY MATERIAL**

**METHODS**

**Data elements extracted from the EHR.**

Each data transfer included selected EHR information looking back to February 1, 2020, allowing for both the collection of information from new patients and follow-up data from individuals already in the cohort who received additional care at the health system. These retrospective updates were accomplished by assigning each patient in the data set from each health system an enduring cryptographically processed Patient ID based on the SHA256 algorithm, which yielded a 64-character unique and private hash-based message authentication code (HMAC). Secure transfer of data from each of the 21 health systems was accomplished via the transfer of data files to a secure SFTP (secure shell [SSH] File Transfer Protocol) portal located at the UW-Madison CEC-UW Coordinating Center. EHR data extraction code was provided via Github to all participating health systems with versions for Oracle-based and SQL-based EHR systems. Extraction code was further customized by the Information Technology (IT) staff at each of 21 health systems to account for health-system-specific EHR structures, tables, formats, and terminologies. The data extraction code was designed to locate and export data elements in a relatively uniform manner across the 21 health systems. The customization of the extraction code (i.e., with system-specific modifications) yielded partly harmonized data files for transfer to the CEC-UW Coordinating with the same basic set of data files and EHR variables within each data file, thus allowing later processing, additional harmonization, and ultimate merging of the data files across all 21 health systems according to a common set of specifications.

This data extraction process yielded five separate “source” data files from each of the 21 health systems formatted as comma-separated-values (CSV) files that were converted to SPSS data files at the CEC-UW Coordinating Center. Processing of the five source SPSS data files included preparation of variables for later analysis with appropriate formatting and labeling as well as computation of new variables such as body mass index (BMI) from weight and height. Further harmonization and merging of data across the 21 health systems were accomplished via computer programs developed by data management staff at the CEC-UW Coordinating Center.

Data elements (variables) were extracted from the EHR via specialized programming code that created five data files containing groups of related data elements. These data files included patient- and encounter-level information on: 1) sociodemographic and health characteristics; 2) pre- and post-COVID ICD-10 diagnoses; 3) clinical encounter data including treatment site (e.g., inpatient, outpatient), encounter-based ICD-10 diagnoses, mortality, ICU admission, intubation, and other clinical data; 4) selected laboratory test results linked to encounters; and 5) selected medications linked to encounters. Not all data types were used in the analyses reported in this paper.

**Weighted Elixhauser Comorbidity Score Calculation.**

Calculation of the Elixhauser Comorbidity Score used ICD-10 diagnoses from the EHR with a 5-year look back pre-COVID-19. The diagnostic groups were identified by using the ICD-10 diagnosis codes listed in Quan et al., "Coding Algorithms for Defining Comorbidities in ICD-9-CM and ICD-10 Administrative Data", Medical Care:43(11), Nov. 2005 p1130-1139. The coding algorithm scans patient diagnostic codes and identifies whether a given ICD-10 code belongs to one or more of 31 different Elixhauser Comorbidity Groups (see S Table1). Each of the Comorbidity Groups is coded as a binary variable where a value of 1 indicates the comorbid condition is present and a value of 0 indicates that the comorbid condition is absent. The total Elixhauser Comorbidity Score is a weighted sum of the 31 binary comorbid conditions, with the weights based on van Walraven et al., “A Modification of the Elixhauser Comorbidity Measures into a Point System for Hospital Death using Administrative Data”, Medical Care, 2009, June 1, pp 626-633 (see Table). The original SAS code for this program was developed by Quan et al at the University of Calgary, Manitoba Centre for Health Policy and modified by CEC-UW data management staff.

**GUIDE Methods.**

A 368-page User Manual with illustrative examples is available at this web address: http://www.stat.wisc.edu/%7Eloh/treeprogs/guide/guideman.pdf. The GUIDE website (https://pages.stat.wisc.edu/~loh/guide.html) has links to downloadable software, datasets, publications, and a six-hour recorded tutorial.

Data are not normalized with GUIDE since the GUIDE algorithm identifies the optimal cut-point in a distribution of naturally occurring scores such as age; therefore, bias is not imposed by non-normal distributions. Imputation was not done with the only variable with missing values in this data set because that variable SDScore was neither missing completely at random (MCAR) nor missing at random (MAR). In addition, GUIDE assigns missings to the node branch with which they share the more similar relationship with the dependent variable. That is, missing status is related to the dependent variable and then the individuals with missing values are assigned to the node value or branch of the known variables that is most similar that relationship. The best selected model was chosen using the GUIDE algorithm defaults, namely, the shortest tree with CV error rate within 1/4 standard error of that of the tree with the smallest CV error rate. Tree feature selection was carried out by the Guide algorithm using chi-squared tests to choose the best variable at every node of the tree.

**Supplemental Tables and Figures**

**S Table 1** – Recent large sample studies on the prediction of COVID-19 mortality

**S Table 2** – Comorbidity groups and associated van Walraven weights used in the calculation of the Elixhauser comorbidity score

**S Table 3** – Pre-Admission vaccine history in the subsample (*N* = 86,732) of those who had received any vaccination (N = 22,799)

**S Table 4** – Descriptive statistics for the subsample of 86,732 COVID-19 patients hospitalized between January 1, 2021 and January 31 2022.

**S Table 5** – Number of patients contributed by each health system to the full sample and subsample

**S Table 6** – Mortality rates as a function of insurance status amongst 145,944 patients hospitalized with COVID-19*.*

**S Fig 1** – Geographic map of the participating healthcare systems

**S Fig 2** – The decision tree generated by a decision tree analysis in the subsample without site as a predictor.

**S Table 1.** *Recent large sample studies on the prediction of COVID-19 mortality*

| **Sample** | **Methods** | **Results** |
| --- | --- | --- |
| **^1^Alballa et al 2021** | | |
| Research studies using machine learning approaches to study two main outcomes: COVID-19 diagnosis and prediction of mortality risk and severity. 52 studies were identified that appeared from 2020 and January 2021 that were focused on readily available clinical and laboratory data as predictors.^1^ | Review of types of machine learning methods used and their features. Sought to evaluate effectiveness of different approaches and especially effective predictors. | Especially effective predictors of COVID-19 severity/mortality were mostly laboratory tests indicating worsening infection (e.g., LDH, lymphocytes, CRP, and renal function values: creatinine, BUN). However, some vital sign measures (e.g., respiratory rate, heart rate), comorbidities (e.g., kidney disease, cancer history, any comorbidity) and demographic measures (age, sex) were especially effective in some models. Authors conclude that it is difficult to determine which of the many machine learning models are most effective. |
| **^2^Baba et al., 2019** | | |
| 56,986 COVID-19 patients in the Japanese COVID-19 Registry were enrolled from March 2, 2020 to February 1, 2022 across 6 epidemiologic waves. Inclusion criteria were (1) a positive COVID-19 test and inpatient treatment.^2^ | Patients were placed into 4 risk groups based on their Coronavirus Clinical Characterization Consortium (*4C*) mortality score^3,4^. Mortality rates were calculated for different age groups and across the wave periods. 4C mortality scores were based on 8 independent parameters at the time of admission: age, sex, number of comorbidities, respiratory rate, peripheral oxygen saturation level, Glasgow Coma Scale score, blood urea nitrogen level, and C-reactive protein level [10]. This study did not examine interactive effects of comorbidities, nor did it examine vaccination as a risk factor. | Risks of mortality changed markedly across time with relatively low rates occurring in the last time period. In general, mortality rates in this Japanese sample were lower than those in early United Kingdom samples. |
| **^5^Bennett et al., 2021** | | |
| A retrospective cohort study, of 174 568 adults with COVID, 32 472 (18.6%) were hospitalized. Data were drawn from 34 medical centers nationwide and were recruited from January 1, 2020 to December 7, 2020. A comparison cohort of 1,133,848 who tested negative for SARS-CoV-2 at the same sites was also recruited.^5^ | Groups were compared over time using multivariable logistic regression while random forest and XGBoost models were used to predict severe clinical course. Patient demographics, comorbidities and clinical tests and values were used to predict COVID-19 mortality and other severity indicators. EHR data were drawn from a national COVID data repository: the National COVID Cohort Collaborative (N3C) | Mortality was 11.6% overall and decreased from 16.4% in March to April 2020 to 8.6% in September to October 2020. A logistic regression model showed that age (odds ratio [OR], 1.03 per year; 95%CI, 1.03-1.04), male sex (OR, 1.60; CI 1.51-1.69), liver disease (OR, 1.20; CI, 1.08-1.34), dementia (OR, 1.26; CI, 1.13-1.41), African American (OR, 1.12; CI, 1.05-1.20) and Asian (OR, 1.33; CI, 1.12-1.57) race, and obesity (OR, 1.36; CI, 1.27-1.46) were independently associated with higher clinical severity (invasive ventilatory support, ECMO [extracorporeal membrane oxygenation], death, or discharge to hospice). The XGBoost and random forest models of severe clinical course showed that model performance varied over regions. Variables that were important in both models included age and multiple laboratory values: oxygen saturation, respiratory rate, blood urea nitrogen, systolic blood pressure, and aspartate aminotransferase. |
| **^6^Hoxha et al., 2023** | | |
| Data were derived from 164 countries on COVID-19 vaccination and mortality of COVID-19 related cases and percent of the population vaccinated. Data were obtained from WHO’s publicly available COVID-19 dashboard as available on 20 September 2022.^6^ | Mortality/1000 cases was the main outcome with vaccination effect captured at both 6 months and 12 months after the first vaccinations in each country. 3 independent variables were used: the percentage of fully vaccinated persons in, 1) the first 6 months, 2) the first 12 months, and 3) the percentage of persons receiving a booster within 12 months. Several country-level variables were used as covariates: GDP per capita, population median age, prevalence of obesity, and overall noncommunicable disease mortality per 100,000 persons. Ordinary least squares regression was used in the context of a log linear model, with models run both with and without covariate adjustment. | Unadjusted regression analysis for the 6-month time period showed that a 10-percentage-point increase in vaccination rates was associated with a 16.0% decrease in COVID-19 mortality (95%CI, 7.9–24.1%). Adjusted analysis showed similar results as did results over the 12-month time period. Vaccination tended to be associated with decreased mortality across different income strata. The unadjusted analysis of booster effects showed that a 10-percentage-point increase in booster rates was associated with a 31.0% decrease in COVID-19 mortality (95%CI, 19.9–42.1%). Again, adjusted analysis showed similar results. |
| **^7^Mahamat-Saleh et al., 2021** |  |  |
| A review and meta-analysis of 186 observational studies (210,447 deaths among 1, 304,587 patients with COVID-19) examining associations between diabetes, hypertension, obesity and smoking and risk of death and estimating the proportion of deaths attributable to these four conditions. Studies identified up to 14 November 2020.^7^ | Random effects models were used to calculate summary relative risks (SRRs). Certainty of evidence was graded with Cochrane methods. There was no attempt to investigate the additive or interactive effects of different comorbidities. Analyses focused on the risk of diabetes, hypertension, obesity, and smoking. | The SRR for mortality of patients with COVID-19 for diabetes = 1.54 (95% CI = 1.44 – 1.64), for hypertension = 1.42 (CI’s = 1.30 – 1.54). The absolute risk of mortality was increased by 14%, 11%, 12%, and 7% for diabetes, hypertension, obesity, and smoking, respectively. |
| **^8^Schwarzinger et al., 2023** |  |  |
| A nationwide retrospective cohort of all adult inpatients discharged with symptomatic COVID-19 from February 24, 2020 to August 28, 2021 in France (N = 465,750).^8^ | Multivariable logistic regression tested associations of pre-existing (past 9 years) mental disorder diagnoses with all-cause mortality. Diagnoses included: dementia, depression, anxiety disorders, schizophrenia, alcohol use disorders, opioid use disorders, Down syndrome, other learning disabilities, and other disorder requiring psychiatric ward admission). Analyses controlled for covariates such as age and physical health comorbidities. Interactions amongst risk factors were not analyzed. Measures and outcomes were gathered from hospital claims data (French National Hospital Discharge database of public and private claims). | 103,890 inpatients (22.3% of the sample) died within 120 days after their first COVID-19 diagnosis date. 153,870 patients (33.0% of the 465,750 patients) had a mental disorder diagnosis. Every mental disorder except for opioid use disorder was independently associated with higher mortality risk. The magnitudes of the adjusted ORs tended to be fairly modest in size (typically with ORs < 1.16). This research also found significantly strong adjusted ORs for a host of other risk factors such as area deprivation, age, male sex, congestive heart failure, liver disease, and renal disease. |

^1^Alballa N, Al-Turaiki I. Machine learning approaches in COVID-19 diagnosis, mortality, and severity risk prediction: A review. Inform Med Unlocked. 2021;24:100564. doi:10.1016/j.imu.2021.100564

^2^Baba, H., Ikumi, S., Aoyama, S., Ishikawa, T., Asai, Y., Matsunaga, N., Ohmagari, N., Kanamori, H., Tokuda, K., Ueda, T., and Kawakami, E. Statistical Analysis of Mortality Rates of Coronavirus Disease 2019 (COVID-19) Patients in Japan Across the 4C Mortality Score Risk Groups, Age Groups, and Epidemiological Waves: A Report From the Nationwide COVID-19 Cohort. Open Forum Infectious Diseases, Volume 10, Issue 1, January 2023, ofac638, <https://doi.org/10.1093/ofid/ofac638>

^3^Asai Y, Nomoto H, Hayakawa K, et al. Comorbidities as risk factors for severe disease in hospitalized elderly COVID-19 patients by different age-groups in Japan. Gerontology 2022; 68:1027–37.

^4^Knight SR, Ho A, Pius R, et al. Risk stratification of patients admitted to hospital with covid-19 using the ISARIC WHO Clinical Characterisation protocol: development and validation of the 4C mortality score. BMJ 2020; 370:m3339.

^5^Bennett TD, Moffitt RA, Hajagos JG, et al. Clinical characterization and prediction of clinical severity of SARS-CoV-2 infection among US adults using data from the US National COVID Cohort Collaborative. JAMA Netw Open. Jul 1 2021;4(7):e2116901. doi:10.1001/jamanetworkopen.2021.16901

^6^Hoxha, I.; Agahi, R.; Bimbashi, A.; Aliu, M.; Raka, L.; Bajraktari, I.; Beqiri, P.; Adams, L.V.Higher COVID-19 Vaccination Rates Are Associated with Lower COVID-19 Mortality: A Global Analysis. Vaccines 2023, 11, 74. https://doi.org/10.3390/vaccines11010074

^7^Mahamat-Saleh Y, Fiolet T, Rebeaud ME, et al. Diabetes, hypertension, body mass index, smoking and COVID-19-related mortality: a systematic review and meta-analysis of observational studies. BMJ Open 2021;11:e052777. doi:10.1136/bmjopen-2021-052777

^8^Schwarzinger M, Luchini S, Teschl M, Alla F, Mallet V, Rehm J (2023) Mental disorders, COVID-19-related life-saving measures and mortality in France: A nationwide cohort study. PLoS Med 20(2): e1004134. https://doi.org/10.1371/journal.pmed.1004134

| **S Table 2.** *Comorbidity groups and associated van Walraven weights used in the calculation of the Elixhauser comorbidity score* | | | |
| --- | --- | --- | --- |
| **Label** | **Comorbidity Group** | **ICD-10 codes** | **Weight** |
| Elix1 | Congestive heart failure | I09.9, I11.0, I13.0, I13.2, I25.5, I42.0, I42.5 - I42.9, I43.x, I50.x, P29.0 | 7 |
| Elix2 | Cardiac arrhythmias | I44.1 - I44.3, I45.6, I45.9, I47.x - I49.x, R00.0, R00.1, R00.8, T82.1, Z45.0, Z95.0 | 5 |
| Elix3 | Valvular disease | A52.0, I05.x - I08.x, I09.1, I09.8, I34.x - I39.x, Q23.0 - Q23.3, Z95.2 - Z95.4 | -1 |
| Elix4 | Pulmonary circulation disorders | I26.x, I27.x, I28.0, I28.8, I28.9 | 4 |
| Elix5 | Peripheral vascular disorders | I70.x, I71.x, I73.1, I73.8, I73.9, I77.1, I79.0, I79.2, K55.1, K55.8, K55.9, Z95.8, Z95.9 | 2 |
| Elix6 | Hypertension, uncomplicated | I10.x | 0 |
| Elix7 | Hypertension, complicated | I11.x - I13.x, I15.x | 0 |
| Elix8 | Paralysis | G04.1, G11.4, G80.1, G80.2, G81.x, G82.x, G83.0 - G83.4, G83.9 | 7 |
| Elix9 | Other neurological disorders | G10.x - G13.x, G20.x - G22.x, G25.4, G25.5, G31.2, G31.8, G31.9, G32.x, G35.x - G37.x, G40.x, G41.x, G93.1, G93.4, R47.0, R56.x | 6 |
| Elix10 | Chronic pulmonary disease | I27.8, I27.9, J40.x - J47.x, J60.x - J67.x, J68.4, J70.1, J70.3 | 3 |
| Elix11 | Diabetes, uncomplicated | E10.0, E10.1, E10.9, E11.0, E11.1, E11.9, E12.0, E12.1, E12.9, E13.0, E13.1, E13.9, E14.0, E14.1, E14.9 | 0 |
| Elix12 | Diabetes, complicated | E10.2 - E10.8, E11.2 - E11.8, E12.2 - E12.8, E13.2 - E13.8, E14.2 - E14.8 | 0 |
| Elix13 | Hypothyroidism | E00.x - E03.x, E89.0 | 0 |
| Elix14 | Renal failure | I12.0, I13.1, N18.x, N19.x, N25.0, Z49.0 - Z49.2, Z94.0, Z99.2 | 5 |
| Elix15 | Liver disease | B18.x, I85.x, I86.4, I98.2, K70.x, K71.1, K71.3 - K71.5, K71.7, K72.x - K74.x, K76.0, K76.2 - K76.9, Z94.4 | 11 |
| Elix16 | Peptic ulcer disease, excluding bleeding | K25.7, K25.9, K26.7, K26.9, K27.7, K27.9, K28.7, K28.9 | 0 |
| Elix17 | AIDS/HIV | B20.x - B22.x, B24.x | 0 |
| Elix18 | Lymphoma | C81.x - C85.x, C88.x, C96.x, C90.0, C90.2 | 9 |
| Elix19 | Metastatic cancer | C77.x - C80.x | 12 |
| Elix20 | Solid tumor without metastasis | C00.x - C26.x, C30.x - C34.x, C37.x - C41.x, C43.x, C45.x - C58.x, C60.x - C76.x, C97.x | 4 |
| Elix21 | Rheumatoid arthritis/collagen vascular diseases | L94.0, L94.1, L94.3, M05.x, M06.x, M08.x, M12.0, M12.3, M30.x, M31.0 - M31.3, M32.x - M35.x, M45.x, M46.1, M46.8, M46.9 | 0 |
| Elix22 | Coagulopathy | D65 - D68.x, D69.1, D69.3 - D69.6 | 3 |
| Elix23 | Obesity | E66.x | -4 |
| Elix24 | Weight loss | E40.x - E46.x, R63.4, R64 | 6 |
| Elix25 | Fluid and electrolyte disorders | E22.2, E86.x, E87.x | 5 |
| Elix26 | Blood loss anemia | D50.0 | -2 |
| Elix27 | Deficiency anemia | D50.8, D50.9, D51.x - D53.x | -2 |
| Elix28 | Alcohol abuse | F10, E52, G62.1, I42.6, K29.2, K70.0, K70.3, K70.9, T51.x, Z50.2, Z71.4, Z72.1 | 0 |
| Elix29 | Drug abuse | F11.x - F16.x, F18.x, F19.x, Z71.5, Z72.2 | -7 |
| Elix30 | Psychoses | F20.x, F22.x - F25.x, F28.x, F29.x, F30.2, F31.2, F31.5 | 0 |
| Elix31 | Depression | F20.4, F31.3 - F31.5, F32.x, F33.x, F34.1, F41.2, F43.2 | -3 |

**S Table 3.** *Pre-Admission vaccine types received in the subsample (N = 86,732) of those who had received any vaccination (N = 22,799)*

| **Vaccine Doses** | **Vaccine 1 Maker** | **Vaccine 2 Maker** | **Vaccine 3 Maker** | **N** | **% of Dose Group** | **% of All Vaccinated Patients** |
| --- | --- | --- | --- | --- | --- | --- |
| 1 | Pfizer |  |  | 2,206 | 39.60 | 9.68 |
|  | Janssen |  |  | 1,914 | 34.36 | 8.40 |
|  | Moderna |  |  | 1,423 | 25.54 | 6.24 |
|  | Unspecified |  |  | 27 | 0.48 | 0.12 |
|  | AstraZeneca |  |  | 1 | 0.02 | 0.00 |
|  | ***Total*** |  |  | 5,571 | 100.00 | 24.44 |
| 2 | Pfizer | Pfizer |  | 8,466 | 62.02 | 37.13 |
|  | Moderna | Moderna |  | 4,834 | 35.42 | 21.20 |
|  | Janssen | Janssen |  | 121 | 0.89 | 0.53 |
|  | Janssen | Pfizer |  | 70 | 0.51 | 0.31 |
|  | Janssen | Moderna |  | 52 | 0.38 | 0.23 |
|  | Unspecified | Unspecified |  | 28 | 0.21 | 0.12 |
|  | Moderna | Pfizer |  | 25 | 0.18 | 0.11 |
|  | Pfizer | Moderna |  | 20 | 0.15 | 0.09 |
|  | Pfizer | Janssen |  | 7 | 0.05 | 0.03 |
|  | Unspecified | Moderna |  | 7 | 0.05 | 0.03 |
|  | Unspecified | Pfizer |  | 6 | 0.04 | 0.03 |
|  | Moderna | Janssen |  | 4 | 0.03 | 0.02 |
|  | AstraZeneca | AstraZeneca |  | 3 | 0.02 | 0.01 |
|  | Moderna | Unspecified |  | 3 | 0.02 | 0.01 |
|  | Sinopharm/BIBP | Sinopharm/BIBP |  | 2 | 0.01 | 0.01 |
|  | Novavax | Novavax |  | 1 | 0.01 | 0.004 |
|  | Unspecified | Janssen |  | 1 | 0.01 | 0.004 |
|  | ***Total*** |  |  | 13,650 | 100.00 | 59.87 |
| 3 | Pfizer | Pfizer | Pfizer | 2,229 | 62.30 | 9.78 |
|  | Moderna | Moderna | Moderna | 1,091 | 30.49 | 4.79 |
|  | Moderna | Moderna | Pfizer | 126 | 3.52 | 0.55 |
|  | Pfizer | Pfizer | Moderna | 74 | 2.07 | 0.32 |
|  | Moderna | Pfizer | Pfizer | 7 | 0.20 | 0.03 |
|  | Unspecified | Moderna | Moderna | 5 | 0.14 | 0.02 |
|  | Unspecified | Unspecified | Pfizer | 5 | 0.14 | 0.02 |
|  | Moderna | Pfizer | Moderna | 4 | 0.11 | 0.02 |
|  | Janssen | Janssen | Pfizer | 3 | 0.08 | 0.01 |
|  | Janssen | Pfizer | Pfizer | 3 | 0.08 | 0.01 |
|  | Moderna | Moderna | Unspecified | 3 | 0.08 | 0.01 |
|  | Pfizer | Pfizer | Unspecified | 3 | 0.08 | 0.01 |
|  | Unspecified | Unspecified | Moderna | 3 | 0.08 | 0.01 |
|  | Unspecified | Unspecified | Unspecified | 3 | 0.08 | 0.01 |
|  | Janssen | Janssen | Moderna | 2 | 0.06 | 0.01 |
|  | Moderna | Unspecified | Unspecified | 2 | 0.06 | 0.01 |
|  | Pfizer | Moderna | Moderna | 2 | 0.06 | 0.01 |
|  | Pfizer | Moderna | Pfizer | 2 | 0.06 | 0.01 |
|  | Pfizer | Pfizer | Janssen | 2 | 0.06 | 0.01 |
|  | Pfizer | Unspecified | Pfizer | 2 | 0.06 | 0.01 |
|  | AstraZeneca | AstraZeneca | Pfizer | 1 | 0.03 | 0.004 |
|  | Janssen | Janssen | Janssen | 1 | 0.03 | 0.004 |
|  | Janssen | Moderna | Moderna | 1 | 0.03 | 0.004 |
|  | Moderna | Janssen | Moderna | 1 | 0.03 | 0.004 |
|  | Moderna | Unspecified | Moderna | 1 | 0.03 | 0.004 |
|  | Pfizer | Janssen | Pfizer | 1 | 0.03 | 0.004 |
|  | Unspecified | Pfizer | Pfizer | 1 | 0.03 | 0.004 |
|  | ***Total*** |  |  | 3,578 | 100.00 | 15.69 |
| *Note:* The full sample includes an additional 19 patients with pre-hospital vaccination; 18 patients had 1 dose (Pfizer *n* = 13, Moderna *n* = 3, Janssen *n* = 2) and 1 patient had two doses (Moderna-Moderna). | | | | | | |

| **S Table 4.** *Descriptive statistics for the subsample of 86,732 COVID-19 patients hospitalized between January 1, 2021 and January 31, 2022.* | | | | |
| --- | --- | --- | --- | --- |
| **Patient Characteristic** | ***N*** | **%** | ***M*** | ***SD*** |
| Elixhauser Comorbidity Index |  |  | 6.05 | 10.13 |
| Age (years) |  |  | 60.04 | 18.62 |
| Social Deprivation Score |  |  | 51.87 | 30.81 |
| Age Groups |  |  |  |  |
| Under 60 Years | 38775 | 44.7 |  |  |
| 60-70 Years | 19593 | 22.6 |  |  |
| Over 70 Years | 28364 | 32.7 |  |  |
| Sex |  |  |  |  |
| Female | 45082 | 52.0 |  |  |
| Male | 41649 | 48.0 |  |  |
| Other | 1 | 0.0 |  |  |
| Race |  |  |  |  |
| American Indian/Alaska Native | 323 | 0.4 |  |  |
| Asian | 2065 | 2.4 |  |  |
| Black or African American | 20800 | 24.0 |  |  |
| Native Hawaiian or Other Pacific Islander | 269 | .3 |  |  |
| White | 53017 | 61.1 |  |  |
| Other Race | 8470 | 9.8 |  |  |
| More Than One Race | 359 | .4 |  |  |
| Missing | 1429 | 1.6 |  |  |
| Ethnicity |  |  |  |  |
| Not Hispanic or Latino | 74222 | 85.6 |  |  |
| Hispanic or Latino | 10901 | 12.6 |  |  |
| Missing | 1609 | 1.9 |  |  |
| Body Mass Index |  |  |  |  |
| Underweight | 2767 | 3.2 |  |  |
| Healthy Weight: | 20100 | 23.2 |  |  |
| Overweight | 24128 | 27.8 |  |  |
| Obese | 28620 | 33.0 |  |  |
| Severely Obese | 10299 | 11.9 |  |  |
| Missing | 818 | .9 |  |  |
| Insurance Status |  |  |  |  |
| Medicare | 43482 | 50.1 |  |  |
| Medicaid | 10790 | 12.4 |  |  |
| Commercial | 24032 | 27.7 |  |  |
| Uninsured | 2046 | 2.4 |  |  |
| Other | 6382 | 7.4 |  |  |
| Rural-Urban Commuting Area |  |  |  |  |
| Rural | 1508 | 1.7 |  |  |
| Small Town | 2871 | 3.3 |  |  |
| Micropolitan Area | 6509 | 7.5 |  |  |
| Metropolitan Area | 75765 | 87.4 |  |  |
| Missing | 79 | 0.1 |  |  |
| Vaccination Status |  |  |  |  |
| No Recorded Vaccination: | 63933 | 73.7 |  |  |
| Yes, At Least One: | 22799 | 26.3 |  |  |
| Vaccination doses |  |  |  |  |
| 0 | 63933 | 73.7 |  |  |
| 1 | 5571 | 6.4 |  |  |
| 2 | 13650 | 15.7 |  |  |
| 3 | 3578 | 4.1 |  |  |

| **S Table 5.** *Number of patients contributed by each health system to the full sample and subsample.* | | | | |
| --- | --- | --- | --- | --- |
| **Health System** | **Full Sample** | | **Subsample** | |
|  | ***N*** | **%** | ***N*** | **%** |
| Site 1 | 1,623 | 1.1 | 1061 | 1.2 |
| Site 2 | 20,739 | 14.2 | 11652 | 13.4 |
| Site 3 | 12,818 | 8.8 | 7046 | 8.1 |
| Site 4 | 3,126 | 2.1 | 2089 | 2.4 |
| Site 5 | 1,703 | 1.2 | 1009 | 1.2 |
| Site 6 | 10,489 | 7.2 | 6881 | 7.9 |
| Site 7 | 2,579 | 1.8 | 1535 | 1.8 |
| Site 8 | 12,741 | 8.7 | 8070 | 9.3 |
| Site 9 | 4,319 | 3.0 | 2817 | 3.2 |
| Site 10 | 1,779 | 1.2 | 941 | 1.1 |
| Site 11 | 494 | 0.3 | 263 | 0.3 |
| Site 12 | 1,1307 | 7.7 | 7672 | 8.8 |
| Site 13 | 10,852 | 7.4 | 5773 | 6.7 |
| Site 14 | 3,157 | 2.2 | 1804 | 2.1 |
| Site 15 | 9,452 | 6.5 | 4388 | 5.1 |
| Site 16 | 5,479 | 3.8 | 3492 | 4.0 |
| Site 17 | 13,444 | 9.2 | 7413 | 8.5 |
| Site 18 | 2,998 | 2.1 | 1703 | 2.0 |
| Site 19 | 2,534 | 1.7 | 1560 | 1.8 |
| Site 20 | 1,035 | 0.7 | 638 | 0.7 |
| Site 21 | 13,276 | 9.1 | 8924 | 10.3 |
| Total | 145,944 | 100.0 | 86,732 | 100.0 |

| **S Table 6.** Mortality rates as a function of insurance status amongst 145,944 patients hospitalized with COVID-19. | | | |
| --- | --- | --- | --- |
| **Insurance Status** | ***N*** | ***N* Died** | **Mortality Rate (95% CI)** |
| Medicare | 75,961 | 9,510 | 0.125 (0.123, 0.128) |
| Medicaid | 17,419 | 815 | 0.047 (0.044, 0.050) |
| Commercial | 38,728 | 1,662 | 0.043 (0.041, 0.045) |
| Uninsured | 3,836 | 443 | 0.115 (0.106, 0.126) |
| Other | 10,000 | 606 | 0.061 (0.056, 0.065) |


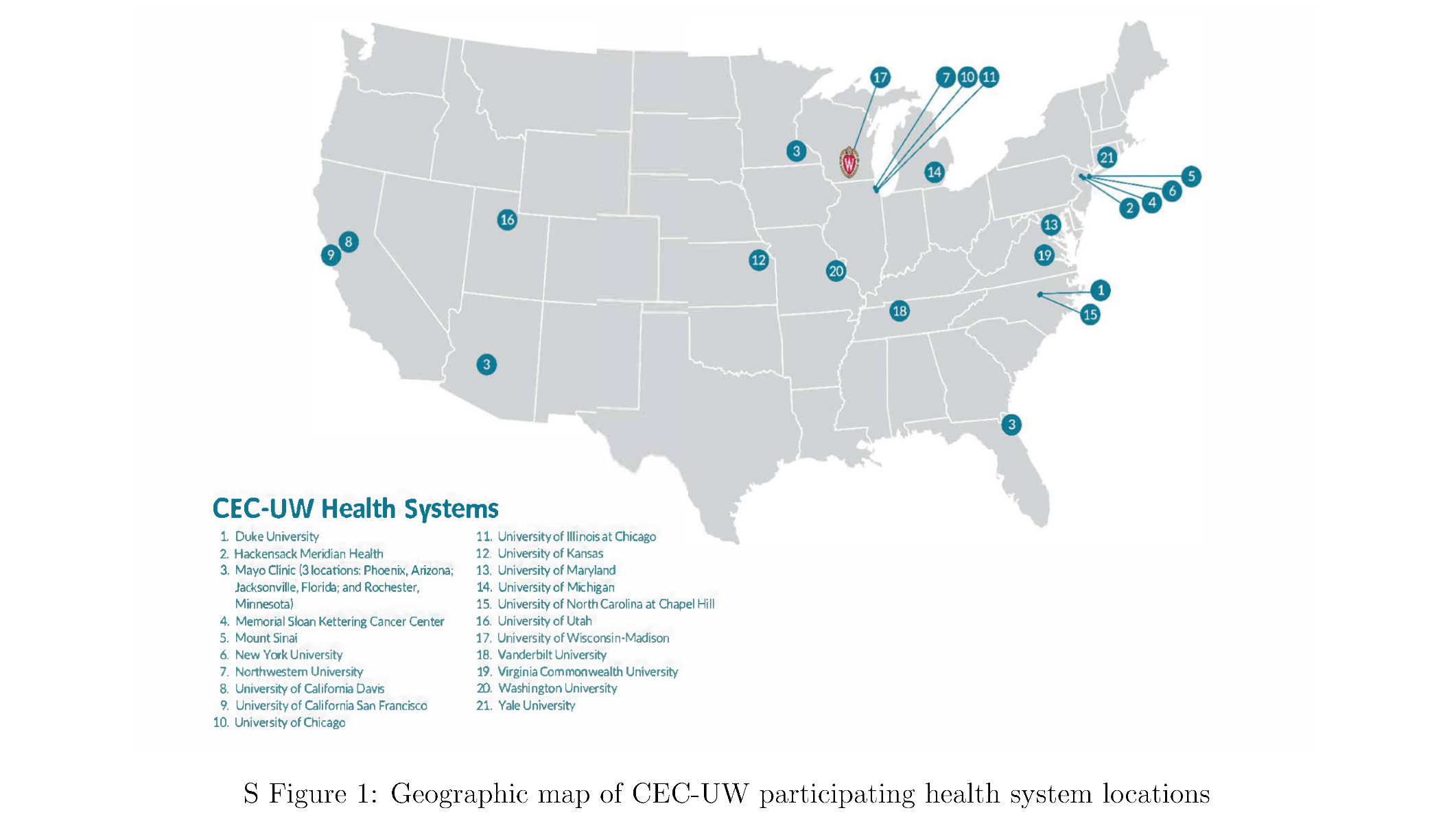


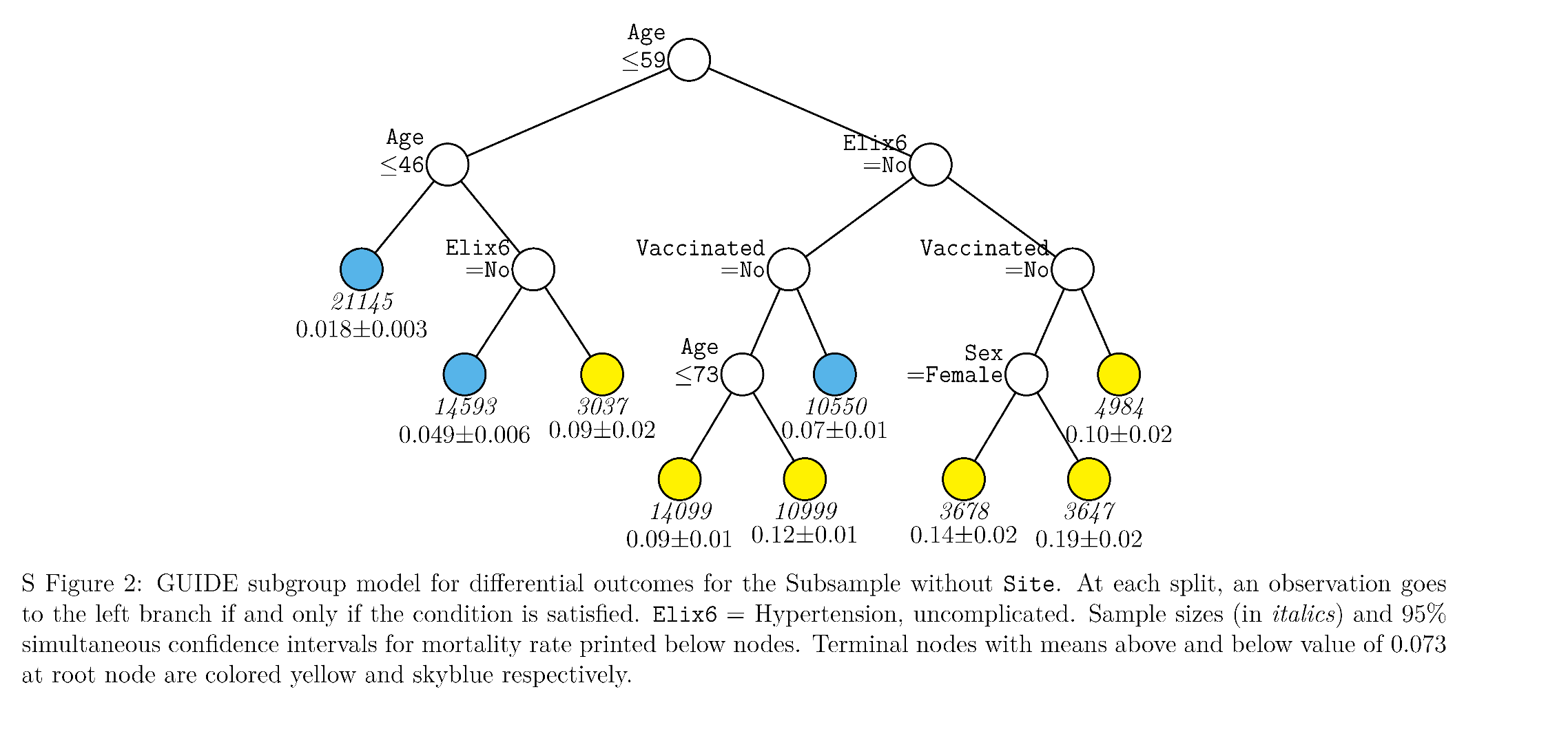

Supplement: Supplementary file 1 — Supplementary Information. [file 41598_2023_31251_MOESM1_ESM.docx]
